# 5G Network Edge Intelligence for Smart Operation and Maintenance of Offshore Wind Power

**DOI:** 10.3390/s26041390

**Published:** 2026-02-23

**Authors:** Yuqing Gao, Lingang Yang, Xialiang Zhu, Congxiao Jiang, Haoyu Wang, Shaonan You, Fangmin Xu

**Affiliations:** 1 PowerChina Huadong Engineering Corporation Limited, Hangzhou 311100, China; gao_yq@hdec.com (Y.G.); yang_lg@hdec.com (L.Y.); zhu_xl@hdec.com (X.Z.); jiang_cx@hdec.com (C.J.); 2 School of Information and Communication Engineering, Beijing University of Posts and Telecommunications, Beijing 100876, China; 17830358268@163.com (H.W.); youshaonan236@163.com (S.Y.)

**Keywords:** offshore wind power intelligent O&M, 5G network deployment, edge intelligence, cloud-edge-terminal collaboration, equipment fault diagnosis, unmanned aerial vehicle inspection, digital twin

## Abstract

As global offshore wind power advances toward deeper, farther waters, harsh Operation and Maintenance (O&M) environments, equipment heterogeneity, and flaws in existing communication (e.g., insufficient 4G bandwidth, high-latency/cost satellite communication) drive the urgent need for intelligent O&M. This paper expounds on the development of Far-Reaching Sea Smart Wind Farms and intelligent service communication demands, studies 5G deployment schemes (hybrid networking, frequency selection, in-turbine coverage, 5G custom networks) and practical cases, discusses core edge intelligence applications (equipment monitoring, inspection, fault diagnosis, digital twin integration), and constructs a “terminal-edge-cloud-network” 5G-edge intelligence integrated architecture. It also summarizes key technology effects, points out current challenges, and looks forward to lightweight large language model deployment at the edge, providing references for 5G edge intelligence implementation in offshore wind power intelligent O&M.

## 1. Background

In recent years, facing increasingly severe greenhouse effects and air pollution, countries worldwide have accelerated the exploration of clean and renewable energy generation. Wind energy, particularly offshore wind energy, as one of the primary clean energy sources, has developed rapidly. Compared to terrestrial wind energy, offshore wind energy resources offer from 20% to 40% higher capacity benefits and possess advantages such as less land occupation, faster operation speeds, higher power output, stable system operation, and being dust-free. As a key focus area for China’s renewable energy development, the scale of offshore wind power has significantly increased. By the end of 2024, the global cumulative installed capacity of offshore wind power reached 83.2 GW, with China’s cumulative installed capacity reaching 41.8 GW, accounting for 50.3% of the global market share, maintaining a leading global position [1]. However, compared to terrestrial wind farms, offshore wind farms are subject to harsh environmental influences such as high humidity, high salt spray, and typhoons, resulting in significantly higher failure rates. The average annual overall failure rate of offshore wind farms is from about 8.5% to 11.8%, while that of terrestrial wind farms is only from 5.2% to 6.9% [2], meaning the offshore failure rate is from 3.3 to 4.9 percentage points higher. In terms of core components, the average annual failure rate of offshore wind turbine blades is about 3.2 occurrences per hundred units, 1.52 times that of terrestrial blades (2.1 occurrences per hundred units) [3]. The failure rate of offshore gearboxes (2.8 occurrences per hundred units) is also 47.4% higher than that of terrestrial ones (1.9 occurrences per hundred units) [4]. Therefore, as the global installed capacity of offshore wind power expands, construction sites are moving towards deeper waters and farther offshore. While O&M demands continuously increase, the O&M environment is becoming harsher, significantly increasing maintenance difficulty. How to improve the efficiency and reduce the cost of offshore wind power O&M has become a core concern for all involved entities.

The growing need for unmanned operations makes utilizing wireless communication technology to guarantee the transmission of O&M information a key technique. The currently prevalent 4G technology on offshore platforms suffers from insufficient bandwidth and coverage gaps, while satellite communication faces challenges of low bandwidth, high latency, high cost, and weak security. Fifth-Generation Mobile Communication Technology (5G), with its high speed, low latency, comprehensive coverage, and network slicing technology, can address data acquisition and wide-area coverage challenges, providing differentiated communication guarantees for industrial control and other services, thereby promoting the digitalization of O&M management.

Furthermore, artificial intelligence (AI) technology combined with wind power O&M application scenarios has led to many new applications, such as blade fault identification and wind power generation prediction. However, centrally deploying AI models in the cloud data center presents challenges like massive data transmission and processing delays [5]. According to Cisco’s estimates, nearly 850 Zettabytes (ZB) of data are generated annually outside the cloud, while global data center traffic remains at 20.6 ZB [6]. Furthermore, empirical data shows that traditional cloud computing typically incurs latencies of from 30 ms to 100 ms, which is intolerable for mission-critical O&M services. In contrast, 5G-enabled edge intelligence can drive these latencies down to less than 10 ms or even 1 ms in specific industrial control scenarios [7,8]. Edge intelligence addresses this by deploying computing capabilities on the edge side of the 5G network [9,10,11]. Its core technologies include task offloading, model compression [12], and collaborative inference [13,14,15].

Integrating 5G with edge intelligence significantly enhances offshore wind power O&M efficiency [7,8]. By deploying lightweight AI models at tower edge nodes, real-time sensor data processing significantly reduces fault detection latency for issues like bolt loosening, eliminating the need to upload massive volumes of raw data. In UAV inspections, cloud-edge collaboration enables real-time high-definition video analysis at the edge—utilizing model pruning and hardware acceleration [16,17]—which drastically shortens response times by transmitting only key features. Furthermore, federated learning [18] enhances gearbox fault detection accuracy in harsh environments while ensuring privacy, and dynamic task offloading balances edge computing resources to provide end-to-end intelligent support.

**Figure 1:**
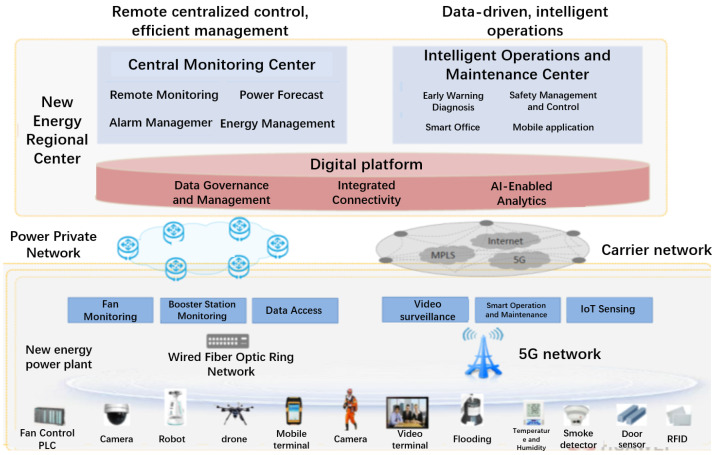
Deployment scheme of 5G intelligent O&M for offshore wind power.

As shown in Figure 1, in current offshore wind farms, equipment related to turbine monitoring, booster station monitoring, and data access converges through the 5G network deployed within the farm, then connects to the centralized monitoring center via submarine wired fiber optic networks. The centralized monitoring center primarily achieves remote centralized control and efficient management through methods like remote centralized monitoring, centralized power prediction, intelligent alarm management, and centralized energy management. The smart O&M center is mainly responsible for O&M management, safety control, efficiency analysis, early warning and diagnosis, smart office operations, and mobile applications.

Although there are many cases of 5G network construction and edge intelligence application in offshore wind power domestically and internationally, there is a lack of universal and mature technical solutions specifically for the 5G network and edge intelligence application in offshore wind power intelligent O&M systems. This paper systematically introduces the typical services of offshore wind power intelligent O&M, along with the deployment technical solutions and application effects of 5G networks synergized with edge intelligence in O&M scenarios, thereby paving the way for subsequent development and technical research.

The subsequent structure of this paper is organized as follows: Section 2 will focus on the 5G edge intelligence-based offshore wind power O&M system architecture and networking scheme, as well as the specific application modes of 5G edge intelligence in scenarios such as equipment condition monitoring, unmanned inspection, turbine fault diagnosis, and digital twin integration. Section 3 will focus on the key technologies of 5G offshore wind power O&M integrated with edge intelligence, including lightweight model compression and acceleration technology, edge computing architecture optimization technology, real-time data processing and analysis technology, etc., combined with practical cases to illustrate the application effects of each technology. Section 4 will systematically sort out the core challenges currently faced by 5G edge intelligence in offshore wind power O&M and analyze the technical nature and impact of each challenge. Section 5 summarizes the core research findings of the full text and provides an outlook for the future, offering references for subsequent technical research and engineering practice.

While several existing reviews have discussed the general potential of 5G and edge computing in industrial IoT, most focus on general architectures and broad application scenarios, leaving a gap in specialized research for offshore wind farms. This paper distinguishes itself by focusing on the system-level integration within unique offshore-specific constraints, such as extreme humidity, salt spray, and long-distance transmission hurdles. The primary contribution of this work lies in the construction of a comprehensive ‘terminal-edge-cloud-network’ architecture specifically optimized for offshore wind O&M. Furthermore, we explicitly highlight the added value of edge intelligence in reducing mission-critical latency and localized data processing, providing a practical technical roadmap for transitioning from traditional manual inspections to autonomous intelligent O&M.

## 2. 5G-Based O&M System Architecture and Networking Scheme

### 2.1. 5G Offshore Wind Power Architecture

**Figure 2:**
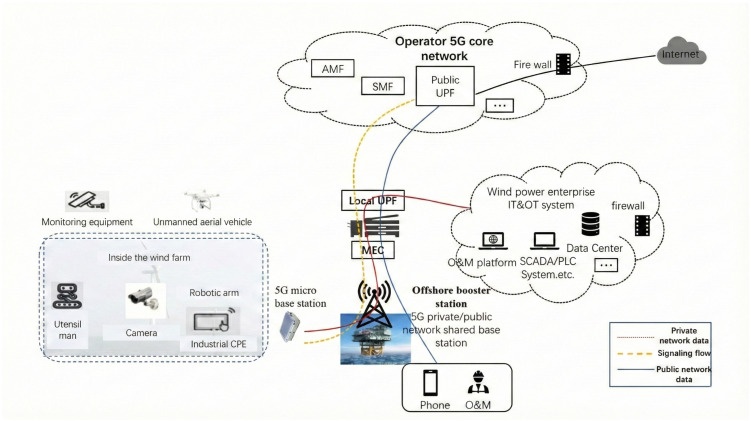
Overall network architecture diagram of 5G hybrid private network for offshore wind power.

To achieve hybrid transmission of multiple services, 5G macro base stations are erected within the wind farm, and 5G small cells are installed in locations with strong signal shielding, such as wind turbine towers and equipment rooms, to ensure comprehensive 5G coverage throughout the farm. The 5G private network is hybrid-networked with the traditional wind power production network. This hybrid networking mode can adapt to densely deployed turbine cluster scenarios through dynamic resource scheduling, ensuring network flexibility. By implementing AI-based handover schemes, research has demonstrated the potential to reduce network handover failure rates by up to 70%, significantly improving connectivity stability for roaming inspection drones and autonomous vessels [8]. Terminal equipment such as inspection robots and sensors can access the edge cloud platform via the 5G private network, with 5G’s large bandwidth ensuring their ability to transmit multimedia data in real-time; dedicated O&M applications can also access the edge cloud platform through specific slices of the 5G private network to obtain required O&M information, supporting intelligent decision-making [19]. The network architecture is shown in Figure 2.

In this networking mode, the onshore centralized control center can collect grid production data, sensor data, video images, and other data in real-time via wired and wireless networks. These data are stored and backed up in the edge cloud on one hand, and on the other hand, they are mapped and calculated based on physical models, with the results sent to the group central cloud at regular intervals. This not only alleviates the transmission bandwidth pressure between the edge cloud and the central cloud but also reduces the data computation and storage pressure on the central cloud.

During network deployment, key factors affecting network coverage in offshore wind power scenarios need to be considered, including PRACH format configuration, frequency selection, equipment and antenna selection, station spacing setting, and deployment methods within turbines. For example, PRACH Format 1 can theoretically achieve 100 km coverage, and low-frequency bands are preferred as the working frequency band for macro base stations.

In terms of networking architecture, wireless coverage within the wind turbine is achieved through STN network + integrated small stations. The equipment of the step-up platform is transmitted back to the equipment in the shore machine room through submarine optical cables, and then connected to the small station network management system above. Through the network management system, it is connected to the 5G core network. The submarine optical cable between the step-up platform and the wind turbine connects the equipment inside the step-up platform and the wind turbine tower, respectively connecting the integrated small station.

The integrated small station system network is mainly composed of integrated small base stations, access gateways, core networks, small base station network management systems, and other network elements. Due to the special structure inside the wind turbine, the signals from the outside of the wind turbine motor cannot penetrate into the interior of the wind turbine motor. Integrated milliwatt-level small base stations can be used. Data can be transmitted back to the small station gateway in the telecommunications machine room through methods such as PON and STN bearer networks. The small station gateway protects data security. Then, it is connected to the 5G core network through the STN bearer network to achieve wireless coverage within the wind turbine.

The offshore wind power communication scenario involves the transmission of services such as HD video surveillance, Unmanned Aerial Vehicle (UAV) inspection, and robot inspection, which have high requirements for large uplink bandwidth, low latency, high reliability, precise positioning, security assurance, and network slicing. A dedicated mode is recommended: the MEC platform should be deployed at the network edge close to the business area [20], adopting the core network UPF sinking method, providing guaranteed low-latency characteristics in network capability, meeting low-latency applications such as remote control of unmanned vessels and UAVs, and significantly improving user experience and data security [7,8].

**Figure 3:**
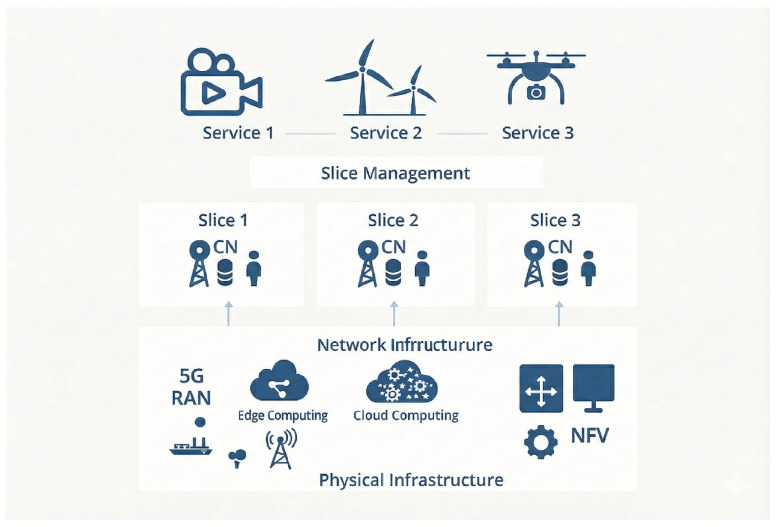
5G custom network solution for offshore wind power O&M.

Based on the above analysis, the 5G private network solution should consider the deployment of professional networks such as wireless networks, transmission networks, and core networks in the UPF sinking mode. The core network user plane (UPF) should be sunk to the telecommunications machine room close to the wind farm to shorten the transmission path and reduce latency. For services such as low-latency control of unmanned aerial vehicle (UAV) inspection, high-definition video transmission of wind turbines, and status monitoring of massive equipment, URLLC, eMBB, and mMTC network slices are, respectively, customized to ensure differentiated service quality. VLAN technology is utilized to divide data such as wind turbine O&M, equipment control, and office management into different logical network domains to achieve isolation and security protection. By integrating the IP RAN protocol, 5G CU/DU separation, and CRAN deployment mode to optimize the transmission and wireless access sides, the communication capability of “low latency, high reliability and specialization” is ultimately achieved, supporting the entire process of intelligent O&M. The network structure is shown in Figure 3.

### 2.2. Applications of 5G with AI in Remote O&M of Offshore Wind Power

Based on the aforementioned 5G network architecture, the integration of artificial intelligence algorithms can be applied to several key aspects of remote O&M scenarios for offshore wind power, as summarized in Table 1. In terms of equipment condition monitoring, sensors deployed inside wind turbine towers and nacelles, combined with intelligent algorithms, enable real-time collection and analysis of data such as equipment vibration, temperature, and stress, allowing for the timely detection of potential equipment faults, such as minor cracks in blades and abnormal wear in gearboxes. In the field of fault diagnosis and predictive maintenance, AI algorithms can accurately diagnose equipment faults and predict the time of fault occurrence in advance using machine learning models based on historical data and real-time monitoring information, facilitating reasonable maintenance scheduling and reducing unplanned downtime. Regarding energy management, AI algorithms can optimize the power output of wind turbines based on real-time information such as wind speed, wind direction, and grid demand, improving energy utilization efficiency. Furthermore, in offshore wind power inspection, Unmanned Aerial Vehicles (UAVs) or unmanned vessels equipped with edge intelligence devices can autonomously plan inspection routes, process captured images and data in real-time, and promptly identify issues such as turbine surface defects and abnormal submarine cable conditions.

#### 2.2.1. AI + UAV Inspection

AI algorithms provide key technical support for UAV-based wind turbine inspection. The core lies in the deep integration of 5G technology and artificial intelligence to address issues in traditional inspection modes such as high data transmission latency, significant cloud computing pressure, and insufficient real-time capability.

UAVs leverage 5G’s large bandwidth and edge node computing to process turbine images and vibration data in real-time, avoiding the latency and bandwidth pressure of raw data uploads. By offloading tasks to the edge UPF, the system enables immediate analysis and shortens the decision-making cycle [21].

Secondly, the deployment of lightweight deep learning models is a key technology. Through optimization techniques like model compression and quantization [22,23,24], UAVs can achieve real-time identification of turbine defects with limited computing resources. For example, Convolutional Neural Networks (CNNs) can be used at edge nodes for rapid detection of faults such as turbine blade cracks and bolt loosening. Edge intelligence model adaptation technology ensures a balance between detection accuracy and computational efficiency.

Furthermore, the cloud-edge-terminal collaborative architecture provides collaborative inference capability for multi-UAV inspections. Through information interaction and task allocation among edge nodes, collaborative inspection path planning and data fusion for multiple UAVs can be achieved [25]. For instance, Multi-Agent Reinforcement Learning (MARL) [8] can be used to optimize the inspection coverage and data interaction strategies of UAV clusters, enhancing inspection efficiency. Additionally, edge intelligence supports privacy-preserving mechanisms based on Federated Learning. In turbine inspection data processing, edge nodes can complete model updates without leaking raw data, meeting the data security requirements of industrial scenarios.

Finally, addressing the constraints of UAV endurance and computing power, edge intelligence employs dynamic task offloading strategies [26]. Complex computational tasks are offloaded to edge nodes or the cloud on-demand, achieving efficient utilization of computing resources. For example, task splitting and offloading algorithms discussed in the literature can dynamically adjust the processing path based on network status and UAV computing power, ensuring the real-time nature and reliability of inspection tasks. The integration of 5G and edge intelligence technologies provides full-process technical support for UAV turbine inspection, from data collection and real-time analysis to decision support.

#### 2.2.2. AI + Robot Tower Inspection

5G with AI addresses the real-time bottleneck in traditional tower inspection through deep coupling of edge computing and artificial intelligence in robot tower inspection and real-time feedback. Specific technical paths include: utilizing the localized computing capability of edge nodes enables robots to process sensor data such as visual images and infrared thermal images in real-time during inspection inside the tower, avoiding control delays caused by uploading raw data to the cloud. For complex tasks, data are transmitted via the internal 5G network to the network edge for proximate analysis of inspection data.

For example, Convolutional Neural Networks are used for rapid detection of faults like weld cracks and bolt loosening inside the tower. Vibration data are collected at high-frequency rates inside the tower, and weld cracks are identified using a CNN-LSTM network. When amplitude exceeding the threshold is detected, the system automatically generates an warning packet and encrypts it for transmission to the remote O&M platform. Experts can remotely control the robot’s mechanical arm to perform bolt tightening via the ultra-low latency 5G channel, ensuring real-time synchronization between remote commands and local perception data.

Relying on the cloud-edge-terminal collaborative architecture, edge nodes interact with the cloud via the 5G communication network. Real-time tasks such as attitude control and path planning are handled at the edge, while complex tasks like tower structural stress analysis are offloaded to the cloud, forming a hierarchical processing model. A Federated Learning mechanism is introduced; after completing local model training at the edge node, each tower inspection robot only uploads model parameters, achieving collaborative model updates while protecting industrial data privacy. Addressing the constraints of robot endurance and computing power, dynamic task offloading strategies adaptively adjust computational task allocation based on device status and network conditions, optimizing resource utilization efficiency.

#### 2.2.3. AI + Turbine Fault Diagnosis

5G with AI plays a key role in the field of turbine fault diagnosis. In the data acquisition and transmission stage, smart sensor networks supporting the IEC 61400-25 standard can comprehensively collect multi-dimensional data such as turbine vibration spectrum, acoustic fingerprint characteristics, and lubricating oil metal content. These sensors can accurately capture early signs of hidden faults, such as micro-cracks in bearings. Simultaneously, data collected by sensors are transmitted via 5G to edge nodes for real-time preprocessing, significantly reducing data analysis processing delay. This avoids network congestion and the processing lag caused by uploading massive volumes of raw data, buying valuable time for subsequent fault diagnosis.

At the level of fault feature extraction and analysis, multiple advanced AI algorithms are integrated to improve performance. On one hand, methods like Variational Mode Decomposition (VMD) are used for time-frequency domain analysis of vibration signals, effectively separating different frequency components and highlighting fault features. For example, it can accurately identify the specific frequency bands where low-frequency bearing faults reside. Feature vectors are compressed and remotely transmitted to the cloud knowledge base. Expert teams use the Federated Learning mechanism to collaboratively optimize the diagnostic model, remotely updating model parameters to substantially improve fault detection accuracy. Edge nodes support remote loading of update packages, achieving a closed loop of “local inference—remote evolution”. On the other hand, by combining the maximum Correlation Kurtosis deconvolution (MCKD) algorithm, the weak fault signal overwhelmed by noise is enhanced through deconvolution operations, and the correlation kurtosis value of the original signal is increased to achieve effective extraction of early fault features. Meanwhile, the Particle Swarm Optimization (PSO) algorithm is used to optimize parameters of models like VMD, ensuring the accuracy and stability of feature extraction.

Edge intelligence focuses on constructing deep neural network models, such as CNN-LSTM hybrids, to learn spatiotemporal features from turbine data and accurately diagnose various faults. For gearbox monitoring, these edge-deployed models provide long-term early warnings for pitting, outperforming traditional threshold methods substantially. Additionally, transfer learning [27] allows these models to adapt to different turbine types significantly faster, enhancing overall system generality.

Additionally, edge intelligence supports adaptive threshold setting based on equipment degradation curves. Compared to fixed threshold methods, sensitivity is increased by 60%, enabling more timely and accurate fault judgment based on changes in turbine operating status. Simultaneously, by constructing a fault relationship knowledge graph with over 200,000 nodes, cross-component associated fault diagnosis is achieved, comprehensively analyzing the causes of turbine faults and avoiding the limitations of single fault diagnosis.

#### 2.2.4. AI + Digital Twin

In offshore wind power intelligent O&M scenarios, the deep integration of GIS technology and Digital Twins is forming a closed-loop management system through edge intelligence. When using GIS technology for vectorization processing of multi-dimensional data such as wind farm wind speed distribution and wind power density, it can rely on lightweight federated learning models in edge intelligence (such as the heterogeneity-aware federated learning framework Helios) to complete terrain adaptability assessment and development priority sorting locally at the edge node, simultaneously constructing the basic geospatial foundation for the Digital Twin. This edge-side intelligent processing mode can significantly reduce raw data backhaul volume, allowing the cloud to focus on the global optimization of the Digital Twin model.

In the links of UAV inspection and turbine layout design, the real-time mapping capability of the Digital Twin is significantly enhanced through edge intelligence: UAV inspection systems based on the 5G MEC architecture can complete defect detection of infrared images in real-time at the flying edge node, simultaneously injecting damage location information into the 3D spatial coordinate system of the Digital Twin model, achieving high-precision fault localization accuracy. In the construction of the turbine Digital Twin, edge-edge collaborative inference technology is adopted. Through intelligent sensor nodes distributed across various layers of the tower, physical signals such as gearbox oil temperature and bearing vibration are collected at high-frequency rates. After preprocessing by a lightweight CNN model, the component status updates of the Digital Twin are driven in real-time, with the visualization latency of key parameters controlled with minimal delay.

The integration of Geographic Information Systems and video surveillance further enhances the dynamic evolution capability of the Digital Twin: Through edge nodes deployed at the booster station, joint reasoning is performed on GIS spatial data and high-definition video streams. Using an early exit mechanism to optimize computational resource allocation [6,28], when anomalies such as blade icing are detected, the edge node can directly trigger three-dimensional warning annotations in the Digital Twin scene. This real-time mapping mode of “physical entity—edge perception—Digital Twin” significantly shortens the health assessment cycle of wind farm equipment compared to traditional methods. Based on the cloud-edge-terminal collaborative computing framework, the global update latency of the Digital Twin model is controlled with minimal delay, providing a technical paradigm of virtual-real interaction for predictive maintenance of offshore wind power.

## 3. Architecture and Key Technologies of 5G Offshore Wind Power O&M Integrated with Edge Intelligence

### 3.1. Integrated Architecture

The integration of edge intelligence and 5G technology has constructed a collaborative integrated technology system of “terminal-edge-cloud-network” for intelligent wind power O&M. In this architecture, 5G networks provide a data transmission foundation for edge intelligence with their high bandwidth and low latency characteristics. For example, data such as wind turbine blade images and vibration spectra collected by lightweight sensors (e.g., millimeter-wave radar and 4K cameras) mounted on drones can be transmitted in real time to edge nodes (e.g., MEC servers [29] deployed on offshore platforms) through 5G URLLC slices, avoiding inspection delays caused by data congestion under traditional 4G networks.

On the other hand, edge intelligence provides key support for the reliable operation and intelligent maintenance of wind turbines in multiple aspects. It preprocesses real-time data such as wind turbine vibration and temperature locally through edge nodes, ensuring ultra-low data processing latency, avoiding transmission delays caused by uploading all raw data to the cloud, gaining time for early fault warning, and significantly improving maintenance real-time performance.

In addition, edge intelligence under the 5G architecture also introduces a federated learning mechanism. Edge nodes of various offshore platforms collaboratively update defect detection models (e.g., FedAvg algorithm) without sharing raw data, which not only meets the data privacy requirements of offshore wind power scenarios but also significantly shortens the model iteration cycle compared to traditional cloud-based schemes. This integrated architecture couples the communication capabilities of 5G networks with the computing capabilities of edge intelligence, realizing the full-process optimization of offshore wind power intelligent O&M from data collection, real-time analysis to decision support.

**Figure 4:**
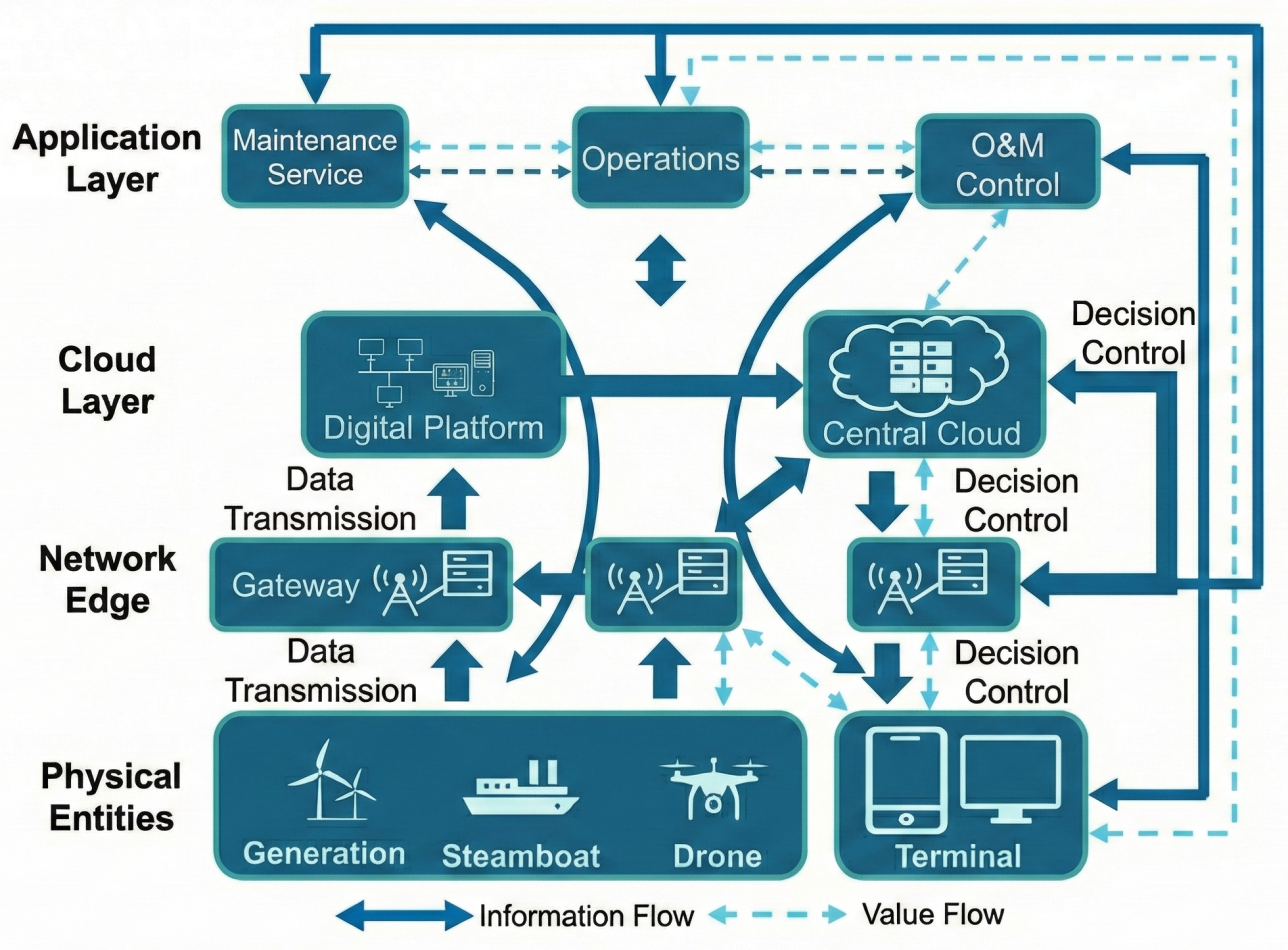
Terminal-edge-cloud-network integrated architecture for offshore wind power O&M.

As shown in Figure 4, in offshore wind power scenarios, 5G base stations serve as key communication hubs to achieve ubiquitous and efficient connection between various equipment in wind farms and O&M systems. Edge computing equipment deployed at locations such as wind turbine towers and step-up stations is equipped with high-performance computing chips and storage capabilities, similar to small data centers, undertaking local data processing tasks.

With the help of lightweight AI algorithm models, edge computing equipment conducts preliminary analysis of data to quickly identify equipment abnormalities, such as blade cracks and loose components. For simple and routine condition monitoring and fault judgment, it can be completed at the edge layer, reducing data upload volume; when encountering complex problems or exceeding the edge processing capacity, the required data are transmitted to the cloud through 5G networks, similar to the heterogeneous task offloading to the cloud layer as shown in the figure, using the powerful computing resources of the cloud for in-depth analysis and decision-making. At the same time, in the terminal-edge-cloud architecture of the entire wind farm, task allocation is dynamically adjusted according to actual needs and network conditions to optimize resource utilization, thereby constructing an efficient and intelligent offshore wind power O&M system and improving the operational reliability and management efficiency of wind farms.

### 3.2. Key Technologies of 5G + Edge Intelligence

#### 3.2.1. Lightweight Model Compression and Acceleration Technology

Lightweight model compression and acceleration technology aims to adapt deep learning models to the limited computing resources of edge devices—characterized by constrained processing power and restricted memory capacity—through model structure optimization and computing paradigm reconstruction. In the offshore environment, these edge devices are often deployed within sealed turbine towers to withstand high salt spray and humidity, which further limits their heat dissipation efficiency. Therefore, model compression is critical not only for resource efficiency but also for ensuring operational reliability under extreme thermal and environmental constraints. This technology takes pruning, quantization, and knowledge distillation [30] as core methods. For example, structured pruning (screening redundant filters based on the L2 norm of the ThiNet algorithm) trims invalid network layers [31] while retaining the model’s feature extraction capability and reducing complexity; low-bit quantization technology maps floating-point parameters to low-precision integers, reduces computational load by combining with hardware acceleration interfaces such as TensorFlow Lite, and compensates for precision loss through dynamic calibration; knowledge distillation [32] enables lightweight models to learn from pre-trained large models. Benchmark studies indicate that a pruned VGG-16 can achieve a 5× weight reduction with negligible error, while ResNet implementations have demonstrated a 2× speedup with only a 1.4% accuracy drop [7]. Additionally, small-footprint DNN frameworks have shown latency improvements of 5–10× by using hash codes, and specific architectural optimizations can achieve an immediate inference speedup of 20% [6,12].

In practical applications, SqueezeNet replaces traditional 3×3 convolutions with 1×1 convolutions, significantly compressing the model parameter volume, achieving accuracy comparable to AlexNet on the ImageNet dataset, and substantially reducing inference latency. MobileNetv3 achieves a highly compressed model size through depthwise separable convolutions and neural architecture search, maintaining high detection accuracy in offshore wind turbine blade crack detection, with inference latency controlled to meet real-time requirements. This effectively resolves the contradiction between the large parameter volume of traditional deep learning [33] models and insufficient edge computing power, avoiding bandwidth pressure and high latency issues caused by uploading massive volumes of raw data to the cloud.

#### 3.2.2. Edge Computing Architecture Optimization Technology

Edge computing architecture optimization technology constructs a hierarchical processing system of “terminal-edge-cloud”, realizing elastic allocation and dynamic scheduling of computing resources through data filtering, task offloading, and cross-layer collaboration. At the data processing level, the terminal layer preprocesses regular data such as environmental parameters and only uploads abnormal features to edge nodes; the edge layer analyzes key data such as equipment vibration spectra in real time to quickly respond to fault warning needs; the cloud focuses on long-term trend modeling and global optimization, reducing the computing load of edge nodes.

In practical applications, task paths are adaptively adjusted based on 5G link quality and edge computing load, retaining real-time tasks such as inspection video analysis at the edge for processing, and offloading complex computations such as tower structure stress analysis to the cloud; at the same time, through edge-edge collaborative reasoning, multiple edge nodes share local features with the help of consensus mechanisms to drive the state update of digital twins. The edge nodes deployed by China Mobile Guangdong at offshore wind power step-up stations adopt a four-layer architecture of “device layer—node layer—fog layer—cloud layer”, preprocessing the vast majority of regular data at the edge, significantly reducing data backhaul volume, and drastically shortening fault warning latency; the hierarchical task processing between MEC servers and terminal equipment also substantially improves the coverage efficiency of multi-drone inspections, effectively breaking the “data sea—decision island” bottleneck of traditional cloud-based centralized processing and solving the transmission latency and resource waste caused by massive volumes of data generated by a single wind turbine per day.

#### 3.2.3. Real-Time Data Processing and Analysis Technology

Real-time data processing and analysis technology integrates signal processing and deep learning, enabling the extraction of equipment fault features in harsh noise environments—particularly the non-stationary noise caused by marine turbulence and wave-induced mechanical vibration—and achieving millisecond-level response anomaly detection.By incorporating environmental-aware filtering, these technologies can effectively distinguish between benign wave-induced movements and actual structural anomalies. This technology plays a role through three core methods: time-frequency domain decomposition, hybrid neural networks, and multi-modal fusion. In terms of time-frequency domain decomposition, variational mode decomposition (VMD) is used to separate multi-frequency band features of vibration signals, and maximum correlation kurtosis deconvolution (MCKD) is combined to enhance weak fault signals, reducing the interference of strong offshore noise on feature extraction; in terms of hybrid neural networks, CNN-LSTM models are adopted to combine spatial feature extraction (e.g., crack morphology) and temporal dependency analysis (e.g., vibration trends) to achieve early fault warning, solving the problem that traditional algorithms are difficult to balance spatial and temporal dimension features; in terms of multi-modal fusion, timestamps of multi-source data such as vibration and infrared are aligned, and fault localization accuracy is improved through feature complementarity, avoiding the limitations of a single data source.

In the application of offshore wind power gearbox fault detection, the VMD and CNN-LSTM hybrid model substantially increases the correlation kurtosis, enabling long-term fault prediction with high accuracy; the DRL-based real-time data processing scheme proposed in “AI-Enhanced Cloud-Edge-Terminal Collaborative Network: Survey, Applications, and Future Directions” dynamically adjusts the data sampling frequency through deep reinforcement learning in the industrial Internet of Things, controlling the vibration signal feature extraction latency with minimal delay, effectively coping with signal interference in offshore wind power environments with strong vibration and high salt spray, and solving the problems of high missed detection rate and large latency of traditional threshold methods.

#### 3.2.4. Cloud-Edge-Terminal Collaboration Technology

Cloud-edge-terminal collaboration technology constructs a cross-layer intelligent collaboration mechanism based on federated learning frameworks and dynamic task scheduling, realizing the balance between data privacy protection and computing efficiency. This collaboration is specifically vital for offshore wind power, as it overcomes the long-distance transmission hurdles of submarine cables by processing mission-critical data at the edge while utilizing the cloud for global trend analysis and model evolution. In terms of federated learning optimization, edge nodes train models locally and only upload gradient information to the cloud for aggregation [34]. This collaborative approach is exceptionally efficient for offshore O&M, as empirical research indicates that 99.9% of gradient exchange in distributed training is redundant [12]. By compressing these redundant gradients, the communication overhead between offshore turbines and the cloud center can be drastically minimized; at the same time, algorithms such as FedAvg are used to optimize cross-node model consistency, but it is necessary to solve the model deviation problem caused by non-independent and identically distributed (Non-IID) data; in terms of dynamic task scheduling, computing tasks are dynamically divided according to network latency and edge computing power—retaining real-time tasks such as inspection image recognition at the edge for processing, and assigning large-scale modeling tasks such as equipment degradation prediction to the cloud to achieve efficient resource allocation; in terms of collaborative reasoning protocols, a three-level collaborative model of “terminal preprocesses data—edge extracts features—cloud makes global decisions” is established, ensuring cross-layer decision consistency through consensus algorithms and avoiding decision conflicts caused by information asymmetry between layers. In the 5G network cooperated by Royal Dutch Shell and KPN, the gearbox fault detection model is collaboratively optimized at edge nodes through the federated learning mechanism, substantially improving the detection accuracy in high salt spray environments; the MADDPG algorithm proposed in “AI-Enhanced Cloud-Edge-Terminal Collaborative Network: Survey, Applications, and Future Directions” coordinates multi-vehicle edge nodes in intelligent transportation, dynamically planning inspection paths through multi-agent reinforcement learning, significantly shortening the task completion time. This technology effectively overcomes the “disconnection failure” and “resource waste” of a single edge or cloud architecture, solving the collaborative decision-making problem in heterogeneous network environments.

#### 3.2.5. Security and Privacy Protection Technology

Security and privacy protection technology constructs a data security and privacy protection system in edge computing environments through hardware protection, encryption protocols, and blockchain technology. This technology adopts a three-layer security architecture: the hardware layer uses IP68 protection and lightweight encryption chips to improve the physical protection capability and data storage security of edge devices in offshore high salt spray environments; the protocol layer integrates federated learning + differential privacy, injecting noise during model training and data transmission to protect data privacy while maintaining model accuracy; the application layer ensures data tamper-proof through blockchain certification and establishes a traceable data trust mechanism. At the same time, the TLS protocol handshake process is optimized, and a certificate verification mechanism suitable for edge devices is designed to reduce the computing load of encryption operations; through privacy computing technologies such as homomorphic encryption, local data desensitization and feature encryption of edge nodes are realized, achieving the goal of “data available but not visible”. The North Sea wind power project in the Netherlands adopts the three-layer protection mechanism in this technology, reducing the risk of data tampering by 90% through blockchain certification, with transaction confirmation time ≤ 5 s; the differential privacy + federated learning scheme proposed in “AI-Enhanced Cloud-Edge-Terminal Collaborative Network: Survey, Applications, and Future Directions” injects Laplacian noise in medical edge computing, improving the model convergence speed by 50% while protecting patient privacy, effectively solving the problems of high computing load and easy data transmission leakage of traditional encryption schemes on edge devices, and balancing security requirements and real-time requirements.

### 3.3. Feasibility Analysis

The practical implementation of the proposed 5G-edge intelligence integrated architecture depends on its economic and operational viability within the harsh offshore environment.

**Economic Feasibility:** While the initial construction of 5G networks involves higher investment costs compared to traditional 4G infrastructure, and the deployment of high-performance MEC servers adds to the hardware procurement expenses, these costs are offset by the long-term reduction in O&M expenditures. Offshore wind farms face significantly higher failure rates (approximately 8.5% to 11.8%) compared to terrestrial wind farms (5.2% to 6.9%) [2]. By utilizing autonomous UAV inspections and early fault diagnosis via edge intelligence, operators can minimize expensive manual offshore maintenance trips and unplanned downtime, which directly lowers the total cost of ownership (TCO) over the turbine’s lifecycle.
**Operational Feasibility:** Conventional communication solutions like 4G and satellite suffer from high costs and insufficient bandwidth. 5G custom networks provide the low-latency channel necessary for real-time monitoring. Furthermore, edge intelligence mitigates the operational burden and bandwidth costs of transmitting massive volumes of raw data to the cloud, significantly reducing backhaul pressure through localized processing.
**System Integration:** The ‘terminal-edge-cloud-network’ architecture provides a practical roadmap. This approach ensures that mission-critical information is analyzed with minimal delay, providing a high-efficiency paradigm for smart wind farm management.

## 4. Existing Problems and Challenges

### 4.1. Dilemma of Dynamic Adaptation Between Lightweight Models and Heterogeneous Edge Resources

In the scenario of edge intelligence deployment in offshore wind power, the dynamic adaptation between lightweight models and heterogeneous edge resources faces prominent challenges. Edge devices generally have significant computing power heterogeneity, with obvious differences not only in processor architectures (e.g., FPGA, GPU [6,35,36,37]) but also in frequent fluctuations in memory capacity. However, existing model compression technologies lack an adaptive adjustment mechanism for the dynamic working conditions of devices, which makes the balance between accuracy and efficiency of lightweight models fundamentally conflict with the actual operating conditions of edge devices, leading to insufficient model generalization ability in complex scenarios (e.g., strong noise environments, temporary insufficient computing power of devices). From a technical perspective, the heterogeneity of hardware instruction sets leads to fragmented model optimization characteristics, and the inference efficiency of the same lightweight model on different edge devices can differ by 3–5 times, greatly increasing the difficulty of large-scale deployment; at the same time, the offshore wind power environment is dynamic. For example, during typhoons, the computing power of devices needs to prioritize core monitoring tasks. Such frequent fluctuations in computing power make it difficult for models to adjust their own complexity in real time. In strong noise scenarios, the model’s ability to extract fault features will be significantly degraded, further affecting the stability of fault detection.

### 4.2. Complexity of Collaborative Scheduling in Cloud-Edge-Terminal Multi-Layer Architecture

Collaborative scheduling of the cloud-edge-terminal multi-layer architecture faces complex challenges. In the hierarchical processing architecture, the time-varying latency of cross-layer data transmission, the dynamic load of edge node computing power, and the multi-objective optimization requirements such as latency, energy consumption, and accuracy are mutually coupled. Traditional scheduling models are mostly designed based on static scenario assumptions, making it difficult to adapt to highly dynamic offshore wind power scenarios (e.g., 5G signal fluctuations affected by typhoons, temporary offline edge nodes), which can easily lead to scheduling strategy failure or resource allocation imbalance. From a technical perspective, multi-objective optimization lacks a unified game model. There is a natural conflict between the terminal’s demand for real-time performance (e.g., millisecond-level fault warning) and the cloud’s goal of global optimization (e.g., trend analysis of long-time series data), which is difficult to balance through a single scheduling strategy; moreover, in distributed training, the distribution differences of fault data from different wind turbines (data heterogeneity) and the communication limitations caused by offshore bandwidth fluctuations will lead to low efficiency of cross-node model aggregation and slow convergence of the global model, seriously affecting the timeliness of O&M decisions.

### 4.3. Technical Bottleneck of Environmental Robustness in Real-Time Data Processing

Real-time data processing of offshore wind power faces technical bottlenecks in environmental robustness. The complex marine environment will cause non-stationary noise interference (e.g., vibration noise caused by typhoons, interference of salt spray on sensors), which is fundamentally inconsistent with the assumptions of existing real-time processing algorithms such as “stationary noise, ideal sampling synchronization”; at the same time, there is a problem of temporal asynchrony in multi-modal data such as vibration, infrared, and voiceprint (differences in sampling frequencies of different sensors, fluctuations in transmission latency, etc.), which will exacerbate the difficulty of feature fusion and lead to a decrease in fault detection accuracy. From a technical perspective, non-Gaussian noise will damage the feature extraction ability of traditional signal decomposition algorithms such as wavelet decomposition, making fault features submerged in noise and difficult to separate effectively; sensor sampling and transmission latency will also lead to spatiotemporal alignment errors of multi-source data (e.g., the timestamp deviation between vibration data and infrared data can reach more than 100 ms), making it impossible to achieve accurate feature complementarity, thereby affecting fault localization accuracy and even causing misjudgment.

### 4.4. Difficulty in Maintaining Consistency of Cross-Layer Collaborative Decision-Making

Maintaining consistency of cross-layer collaborative decision-making faces many challenges. In the distributed training framework, local edge learning and cloud model aggregation face a non-linear trade-off between “communication efficiency—model accuracy” in network unstable scenarios such as offshore 5G signal interruption and sudden bandwidth drop—frequent transmission of model parameters to ensure accuracy will increase communication overhead; reducing transmission frequency to improve efficiency will lead to an expansion of model deviation between edge and cloud. At the same time, the priority conflict of multi-layer decision logic (e.g., local fault warning at the edge and global O&M planning at the cloud) lacks a standardized arbitration mechanism, which can easily lead to decision conflicts. From a technical perspective, network jitter will lead to the accumulation of model aggregation errors. Especially in data heterogeneous scenarios, the local models of edge nodes are significantly different from the global cloud models, with degraded generalization ability, making it difficult to adapt to the O&M needs of different wind farms; in addition, there is information asymmetry in cross-layer decision-making (e.g., edge nodes determine the need for emergency shutdown based on real-time data, while the cloud believes it can continue to operate based on long-term trends), and there is no unified state synchronization mechanism to quickly resolve such conflicts, seriously affecting the efficiency of O&M responses.

### 4.5. Essential Conflict of Resource Competition in Lightweight Security Mechanisms

Lightweight security mechanisms face an essential conflict of resource competition. The demand for security protection (e.g., encrypted transmission, privacy protection) of edge devices has a natural resource competition relationship with computing efficiency. Although existing lightweight security schemes have simplified encryption algorithms, they still need to occupy 15–30% of CPU resources for cryptographic operations under the limited computing power of edge devices, which forms an irreconcilable contradiction with the performance requirements of real-time AI inference (e.g., fault detection models need to occupy 40–60% of the CPU), leading to increased model inference latency or degraded security protection. From a technical perspective, the CPU resource exclusivity between cryptographic operations and AI inference is significant. When the two run simultaneously, resource contention is likely to occur, resulting in a high proportion of security overhead and affecting the real-time performance of O&M tasks; in addition, there is a physical conflict between hardware protection design (e.g., encryption chips) and heat dissipation requirements—in the offshore high humidity and high salt environment, the heat dissipation efficiency of encryption chips decreases, which is prone to triggering equipment overheating protection, exacerbating the operational reliability risk of equipment, and further limiting the deployment of security mechanisms.

## 5. Conclusions and Prospects

The deep integration of 5G technology and edge intelligence provides an innovative technical paradigm for offshore wind power intelligent O&M. This paper systematically sorts out the development trends of global far-reaching sea smart wind farms and the communication needs of intelligent O&M services, conducts in-depth research on 5G deployment schemes and domestic and foreign practical cases in offshore wind power, discusses the core applications of edge intelligence in equipment condition monitoring, drone/robot inspection, wind turbine fault diagnosis, and digital twin integration, constructs a “terminal-edge-cloud-network” 5G-edge intelligence integrated architecture, summarizes the application effects of key technologies, and analyzes the current technical challenges. In the future, the lightweight deployment of large language models (LLMs) [38] at the edge can be explored.Expected 6G performance metrics suggest that spectrum efficiency will be from 1.5 to 3 times higher than 5G, with energy efficiency increasing by up to 20 times, providing an ultra-sustainable foundation for pervasive edge intelligence [8].

## Figures and Tables

**Table 1:** Applications of artificial intelligence in offshore wind power O&M scenarios.

Application Scenario	Core Technology Support	Key Effects
Equipment Condition Monitoring	Wind turbine vibration/temperature/stress sensors; Lightweight edge AI models (e.g., MobileNet); Local data preprocessing	Real-time identification of minor blade cracks and abnormal gearbox wear; Significantly reduces the volume of routine data uploads, lowering bandwidth pressure.
Fault Diagnosis and Predictive Maintenance	Machine learning models; Cloud-edge collaborative iteration; VMD/MCKD signal processing algorithms	Accurate diagnosis of faults such as gearbox tooth surface pitting; Provides long-term early warnings; Substantially improves detection accuracy in harsh environments.
UAV Inspection	Edge-based real-time high-definition video processing; Lightweight AI models; Federated learning for privacy protection	Drastically reduces fault response times; Avoids uploading massive volumes of raw data; Identifies blade cracks and submarine cable anomalies.
Robot Tower Inspection	CNN-LSTM hybrid model (High-frequency vibration data acquisition); Ultra-low latency 5G channel	Detects weld cracks and bolt loosening; Supports remote robotic arm maintenance; Eliminates delay caused by raw data upload.
Digital Twin Integration	Lightweight federated learning (Helios framework); High-frequency physical signal acquisition; STN edge node (GIS + high-definition video inference)	Real-time visualization of twin parameters; Equipment health assessment cycle significantly shortened; High-precision fault localization.

## Data Availability

The data presented in this study are available on request from the corresponding author.
